# Supplementary material for: Neural Networks in the Design of Molecules with Affinity to Selected Protein Domains
Source: Int J Mol Sci. 2023 Jan 16;24(2):1762. doi: 10.3390/ijms24021762 (PMC9865393; doi:10.3390/ijms24021762)
Supplement: Supplementary file 1 [file ijms-24-01762-s001.zip › Equations.pdf]

Equations and methods

Equation S1. Quantitative estimation of Drug-Likeness [1] (see **File S47**)

$$QED_w = \exp\left[\frac{W_{MW}\ln(d_{MW})+W_{ALOGP}\ln(d_{ALOGP})+W_{HBA}\ln(d_{HBA})+W_{HBD}\ln(d_{HBD})+W_{PSA}\ln(d_{PSA})+W_{ROTB}\ln(d_{ROTB})+W_{AROM}\ln(d_{AROM})+W_{ALERTS}\ln(d_{ALERTS})}{W_{MW}+W_{ALOGP}+W_{HBA}+W_{HBD}+W_{PSA}+W_{ROTB}+W_{AROM}+W_{ALERTS}}\right]$$

, where W_x means weight of natural logarithm of each molecular descriptor, for MW weight is 0.66 then - * $\ln(d_{MW})$, ALOGP weight is 0.46 then - *$\ln(d_{ALOGP})$, HBA weight is 0.05 * $\ln(d_{HBA})$, HBD weight is 0.61 then - * $\ln(d_{HBD})$, PSA weight is 0.06 then - * $\ln(d_{PSA})$, ROTB weight is 0.65 then - * $\ln(d_{ROTB})$, AROM weight is 0.48 then - * $\ln(d_{AROM})$, ALERTS weight is 0.95 then - * $\ln(d_{ALERTS})$. Functions of desirability for each molecular descriptor:

$$d_x = a + \frac{b}{\left[1+\exp\left(-\frac{x-c+\frac{d}{2}}{e}\right)\right]}\left[1-\frac{1}{1+\exp\left(-\frac{x-c-\frac{d}{2}}{f}\right)}\right]$$

$$d_{MW} = 2.817 + \frac{392.575}{\left[1+\exp\left(-\frac{MW-290.749+\frac{2.420}{2}}{49.223}\right)\right]}\left[1-\frac{1}{1+\exp\left(-\frac{MW-290.749-\frac{2.420}{2}}{65.371}\right)}\right]$$

$$d_{ALOGP} = 3.173 + \frac{137.862}{\left[1+\exp\left(-\frac{ALOGP-2.535+\frac{4.581}{2}}{0.823}\right)\right]}\left[1-\frac{1}{1+\exp\left(-\frac{ALOGP-2.535-\frac{4.581}{2}}{0.576}\right)}\right]$$

$$d_{HBA} = 2.949 + \frac{160.461}{\left[1+\exp\left(-\frac{HBA-3.615+\frac{4.436}{2}}{0.290}\right)\right]}\left[1-\frac{1}{1+\exp\left(-\frac{HBA-3.615-\frac{4.436}{2}}{1.301}\right)}\right]$$

$$d_{HBD} = 1.619 + \frac{1010.051}{\left[1+\exp\left(-\frac{HBD-0.985+\frac{10^{-9}}{2}}{0.714}\right)\right]}\left[1-\frac{1}{1+\exp\left(-\frac{HBD-0.985-\frac{10^{-9}}{2}}{0.921}\right)}\right]$$

$$d_{PSA} = 1.877 + \frac{125.223}{\left[1+\exp\left(-\frac{PSA-62.908+\frac{87.834}{2}}{12.020}\right)\right]}\left[1-\frac{1}{1+\exp\left(-\frac{PSA-62.908-\frac{87.834}{2}}{28.513}\right)}\right]$$

$$d_{ROTB} = 0.010 + \frac{272.412}{\left[1+\exp\left(-\frac{ROTB-2.558+\frac{1.566}{2}}{1.272}\right)\right]}\left[1-\frac{1}{1+\exp\left(-\frac{ROTB-2.558-\frac{1.566}{2}}{2.758}\right)}\right]$$

$$d_{AROM} = 3.218 + \frac{957.737}{\left[1+\exp\left(-\frac{AROM-2.275+\frac{10^{-9}}{2}}{1.318}\right)\right]}\left[1-\frac{1}{1+\exp\left(-\frac{AROM-2.275-\frac{10^{-9}}{2}}{0.376}\right)}\right]$$

$$d_{ALERTS} = 0.010 + \frac{1199.094}{\left[1+\exp\left(-\frac{ALERT+0.090+\frac{10^{-9}}{2}}{0.186}\right)\right]}\left[1-\frac{1}{1+\exp\left(-\frac{ALERT+0.090-\frac{10^{-9}}{2}}{0.875}\right)}\right]$$

Each of d_x value is then compared to the maximal possible d_{max}. The results are used during final QED calculations.

Example: QED for aspirin: MW = 180.16 g/mol; ALOGP = 1.31; HBA = 4; HBD = 1; PSA = 63.6 Å²; ROTB = 3, AROM = 1; ALERTS = 2.

$$QED_{aspirin} = \exp\left[\frac{0.66\ln(0.337)+0.46\ln(0.846)+0.05\ln(0.886)+0.61\ln(0.986)+0.06\ln(0.976)+0.65\ln(0.992)+0.48\ln(0.827)+0.95\ln(0.241)}{0.66+0.46+0.05+0.61+0.06+0.65+0.48+0.95}\right] = 0.56$$

Equation S2. Lipinski's rule of 5 [2]

Molecular descriptors set – Molecular weight – MW, octanol-water partition coefficient – LogP, number of hydrogen donors – HBD, number of hydrogen acceptors – HBA, number of rotatable bonds – ROTB.

To pass this test molecule should fulfill the below conditions:

$$MW \leq 500\ g/mol, LogP \leq 5, HBD \leq 5, HBA \leq 10, ROTB \leq 5$$

Example: Aspirin: MW = 180.16 g/mol; LOGP = 1.31; HBA = 4; HBD = 1; ROTB = 3

MW(aspirin) < 500, LogP < 5, HBA <10, HBD <5, ROTB < 5 Aspirin passes this filtration.

Equation S3. Normalization function

$$\text{Normalized value} = \frac{\text{value} - \text{minimal value in dataset}}{\text{maximal value in dataset} - \text{minimal value in dataset}}$$

Example: six elements are given 3, 8, 15, 32, 12, 45.

Normalization results are:

$$\text{Normalized value}(3) = \frac{3-3}{45-3} = 0.000 \qquad \text{Normalized value}(32) = \frac{32-3}{45-3} = 0.690$$

$$\text{Normalized value}(8) = \frac{8-3}{45-3} = 0.119 \qquad \text{Normalized value}(12) = \frac{12-3}{45-3} = 0.214$$

$$\text{Normalized value}(15) = \frac{15-3}{45-3} = 0.286 \qquad \text{Normalized value}(45) = \frac{45-3}{45-3} = 1.000$$

Equation S4. The binding free energy calculation [3]

$\Delta G = \left(V^{L-L}_{bound} - V^{L-L}_{unbound}\right) + \left(V^{P-P}_{bound} - V^{P-P}_{unbound}\right) + \left(V^{P-L}_{bound} - V^{P-L}_{unbound} + \Delta S_{conf}\right)$, where L refers to ligand and P indicates protein in the docking energy calculation.

Each V parameter has a unit of kcal/mol.

ΔS_{conf} - the conformational entropy lost upon binding.

Equation S5. Energetic terms calculation [3]

$$V = W_{vdw}\sum_{i,j}\left(\frac{A_{ij}}{r_{ij}^{12}} - \frac{B_{ij}}{r_{ij}^{6}}\right) + W_{hbond}\sum_{i,j}E(t)\left(\frac{C_{ij}}{r_{ij}^{12}} - \frac{D_{ij}}{r_{ij}^{10}}\right) + W_{elec}\sum_{i,j}\frac{q_i q_j}{e(r_{ij})r_{ij}} + W_{sol}\sum_{i,j}(S_iV_j + S_jV_i)e^{\left(\frac{-r_{ij}^2}{2\sigma^2}\right)}$$

Where W_{vdw} means weighting constant for Van der Waals interactions, $A_{ij} = 4\epsilon\sigma^{12}$ [4] (ϵ means strength of attraction by particles, σ means van der Waals radius (equals ½ of the internuclear distance between nonbonding particles), $B_{ij} = 4\epsilon\sigma^{6}$ [4], r is the distance of separation between both particles (from one center of the particle to the center of another particle). W_{hbond} means weighting constant for hydrogen-bonding. W_{elec} means weighting constant for electrostatics. q means the charge [4].
W_{sol} means weighting constant for desolvation.

First-term, $W_{vdw}\sum_{i,j}\left(\frac{A_{ij}}{r_{ij}^{12}} - \frac{B_{ij}}{r_{ij}^{6}}\right)$, is a typical 6/12 potential for dispersion/repulsion interactions [5, 3], the Lennard-Jones Potential [4].

Second, $W_{hbond}\sum_{i,j}E(t)\left(\frac{C_{ij}}{r_{ij}^{12}} - \frac{D_{ij}}{r_{ij}^{10}}\right)$, describes input from H-bond based on 10/12 potential. Parameters C and D are assigned to give the maximal energy outcome for hydrogen-oxygen and nitrogen H-bonds, which is about 5 kcal/mol at 1.9 Å length and with an energy of about 1 kcal/mol when an H-bond with sulfur is formed at 2.5 Å in length. Function E(t) provides energy change based on the angle t from ideal H-bonding geometry [5, 3].

Third term, $W_{elec}\sum_{i,j}\frac{q_i q_j}{e(r_{ij})r_{ij}}$, refers to the screening Coulomb potential in electrostatics [5, 3, 4].

Fourth, $W_{sol}\sum_{i,j}(S_iV_j + S_jV_i)e^{\left(\frac{-r_{ij}^2}{2\sigma^2}\right)}$, so-called desolvation potential, which is calculated on the volume of atoms (V) that surround a certain atom and shelter it from solvent, the S parameter is used there as a weight. Also, an exponential term can be found. It is related to distance-weighting input and is given by σ, and equals 3.5 Å [5, 3].

Equation S6. Categorical cross-entropy equation [6] (see **File S47**)

Loss $= -\sum_{i}^{\text{output size}} y_i * \log(\hat{y_i})$, where $\hat{y_i}$ is i-th scalar value in the model output, y_i is the corresponding target value, and output size (classes) is the number of scalar values in the model output.

Table 1. Categorical cross-entropy exemplary calculations.

Target values	model output	Loss	Partial loss
Structure 1	Structure 1	Structure 1	
0	0.110	0.418	0.000
1	0.720		-0.143
1	0.530		-0.276
0	0.011		0.000
Structure 2		Structure 2	
1	0.560	0.293	-0.252
0	0.240		0.000
1	0.910		-0.041
0	0.110		0.000
Structure 3		Structure 3	
0	0.180	0.077	0.000
1	0.930		-0.032
1	0.920		-0.036
1	0.980		-0.009
Structure 4		Structure 4	
0	0.050	0.036	0.000
0	0.020		0.000
1	0.950		-0.022
1	0.970		-0.013

Method S1. SYBA classifier – a SYnthetic Bayesian Accessibility classifier is a tool that classifies organic compounds as easy-to-synthesize (ES) or hard-to-synthesize (HS). This algorithm is a fragment-based method. The analyzed molecule is decomposed into ECFP4-like fragments, and a score is assigned to each fragment. All scores are then summed. If the resultant score is positive, the structure is considered as easy-to-synthesize [7].

Each compound is represented by a binary fingerprint $F = [f_1, f_2, f_3, \ldots, f_M]$ of length M. f_i indicates the presence (1) or absence (0) of the specific fragment i in the compound. This fingerprint is used to assign the molecule to a class $C \in \langle ES, HS \rangle$. The Bayesian theorem is used $p(C|F) = \frac{p(F|C)p(C)}{p(F)}$, where p(C|F) is the posterior probability that a compound with a certain set of molecular fragments F belongs to class C. The likelihood p(F|C) is the conditional probability that a compound from the class C contains a set of molecular fragments F. The marginal probabilities p(F) and p(C) express our belief to see a set of molecular fragments F and the molecule that belongs to the class C.

SYBA score is calculated by use of the equation shown below [8].

$$\text{SYBA}(F) = \sum_{i=1}^{M} \ln\left(\frac{p(f_i|ES)}{p(f_i|HS)}\right)$$

Method S2. LSTM cell from a mathematical point of view. [9] (see **Figure 1**)

Three types of gates are distinguished: input, forget and output gate. All they are sigmoid $\left(\mathrm{sigmoid(t)} = \frac{1}{1+e^{-t}}\right)$, activation functions, so the output is in the range from 0 to 1. This is used to fulfill the necessity of positive output; this is due to fact that the answer is whether the particular feature should be kept or discarded. Zero as a result blocks the gate and one allows passing information through it.

The equations for each gate are given below:

$i_t = \delta(w_i[h_{t-1}, x_t] + b_i)$ – for the input gate, the latest information to be stored,

$f_t = \delta(w_f[h_{t-1}, x_t] + b_f)$ – for the forget gate, throwing away information from the cell,

$o_t = \delta(w_o[h_{t-1}, x_t] + b_o)$ – for the output gate, activation supplying and final output creation at given timestamp "t"

δ – stands for sigmoid function, w_x – weight for the respective gate(x), h_{t-1} – output of the previous LSTM block, x_t – input at the current timestamp, b_x – biases for respective gate

Equations for the cell state, candidate cell state, and final output:

$\tilde{c}_t = \tanh(w_c[h_{t-1}, x_t] + b_c)$; $\tilde{c}_t$ – candidate for cell state at timestamp

$c_t = f_t * c_{t-1} + i_t * \tilde{c}_t$; c_t – cell state at timestamp

$h_t = o_t * \tanh(c_t)$; h_t - hidden state at timestamp

To get the output SoftMax activation is applied: $\mathrm{Output} = \mathrm{softmax}(h_t)$

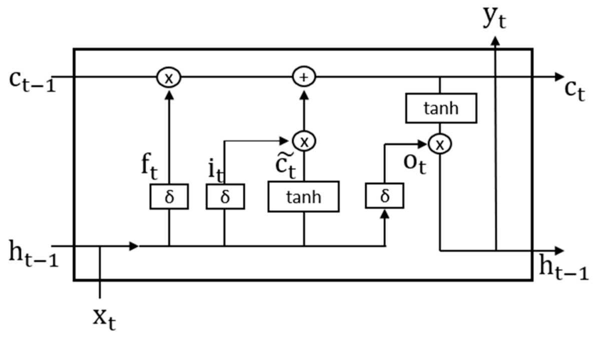

Figure 1. The LSTM scheme.

Method S3. Tanimoto similarity [10] (see **File S47**, see **Figure 2**)

Computation is done as an inverse of the distance of descriptor space measurement. For purpose of this work, molecular fingerprints are compared.

$\mathrm{Tc(A, B)} = \frac{c}{a+b-c}$, where a and b are representing several features present in compounds A and B and c is the number of features that are common for both.

This means that in the case of fingerprints usage feature means on-bits numbers similarly to arrays used when molecular sequences are transformed into numerical arrays.

Output is given in the range from 0 to 1.

Structures are considered to be similar if T > 0.85, but this does not give information about possible similar bioactivity, this parameter depends on many more variables.

Should be mentioned that one as the outcome does not necessarily mean that our structures are identical, it means that they have the same fingerprints.

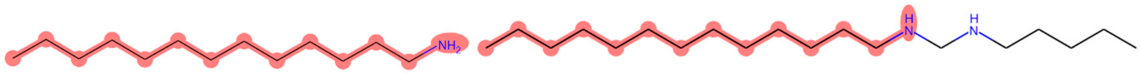

Figure 2. Similarity between two structures; tridecan-1-amine and [(pentylamino)methyl](tridecyl)amine.

Structures are transformed into molecular objects (RDkit library [11]) and corresponding fingerprints are created then they are compared, and the result is $\mathrm{Tc(A, B)} = 0.(44)$.

Method S4. Molecular docking visualization [1]

In the figure shown below (see **Figure 3**) the search space for molecular docking is visualized. This is where the ligand will be attached to the macromolecule. The Lamarckian genetic algorithm is used there [12].

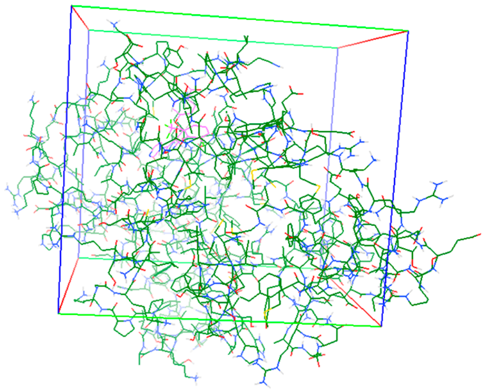

Figure 3. Search space in molecular docking for 7NPC with native ligand visualization, inside which grids are calculated and used by genetic algorithm which searches for the best ligand pose.

References

1. Bickerton, G. Richard, Gaia V. Paolini, Jérémy Besnard, Sorel Muresan, and Andrew L. Hopkins. "Quantifying the Chemical Beauty of Drugs." Nature Chemistry 4, no. 2 (January 24, 2012): 90–98. https://doi.org/10.1038/nchem.1243
2. Lipinski, C. A., F. Lombardo, B. W. Dominy, and P. J. Feeney. "Experimental and Computational Approaches to Estimate Solubility and Permeability in Drug Discovery and Development Settings." Advanced Drug Delivery Reviews 46, no. 1–3 (March 1, 2001): 3–26. https://doi.org/10.1016/s0169- 409x(00)00129-0
3. AutoDock UserGuide https://autodock.scripps.edu/wp-content/uploads/sites/56/2021/10/AutoDock4.2.6_UserGuide.pdf [15.02.2022]
4. Atkins, P. W., and Julio De Paula. *Physical Chemistry for the Life Sciences*. Oxford, UK : New York: Oxford University Press ; W.H. Freeman, 2006.
5. Morris, Garrett M., Ruth Huey, William Lindstrom, Michel F. Sanner, Richard K. Belew, David S. Goodsell, and Arthur J. Olson. "AutoDock4 and AutoDockTools4: Automated Docking with Selective Receptor Flexibility." *Journal of Computational Chemistry* 30, no. 16 (December 2009): 2785–91. https://doi.org/10.1002/jcc.21256.
6. Categorical cross-entropy: https://peltarion.com/knowledge-center/documentation/modeling-view/build-an-ai-model/loss-functions/categorical-crossentropy [21.03.2022]
7. Voršilák, Milan, and Daniel Svozil. "Nonpher: Computational Method for Design of Hard-to-Synthesize Structures." Journal of Cheminformatics 9, no. 1 (March 20, 2017): 20. https://doi.org/10.1186/s13321-017-0206-2.

8. Voršilák, Milan, Michal Kolář, Ivan Čmelo, and Daniel Svozil. “SYBA: Bayesian Estimation of Synthetic Accessibility of Organic Compounds.” Journal of Cheminformatics 12, no. 1 (December 2020): 35. https://doi.org/10.1186/s13321-020-00439-2.
9. Brownlee, J. Long Short-Term Memory Networks with Python: Develop Sequence Prediction Models with Deep Learning. Jason Brownlee, 2017
10. Maggiora, Gerald, Martin Vogt, Dagmar Stumpfe, and Jürgen Bajorath. “Molecular Similarity in Medicinal Chemistry: Miniperspective.” Journal of Medicinal Chemistry 57, no. 8 (April 24, 2014): 3186–3204. https://doi.org/10.1021/jm401411z.
11. RDKit: Open-source cheminformatics; http://www.rdkit.org [01.02.2022]
12. Ross, Brian. “A Lamarckian Evolution Strategy for Genetic Algorithms.” In *Practical Handbook of Genetic Algorithms*, edited by Lance Chambers. CRC Press, 1998. https://doi.org/10.1201/9781420050080.ch1.
