# Supplementary material for: Neural Networks in the Design of Molecules with Affinity to Selected Protein Domains
Source: Int J Mol Sci. 2023 Jan 16;24(2):1762. doi: 10.3390/ijms24021762 (PMC9865393; doi:10.3390/ijms24021762)
Supplement: Supplementary file 1 [file ijms-24-01762-s001.zip › Figures.pdf]

Figures

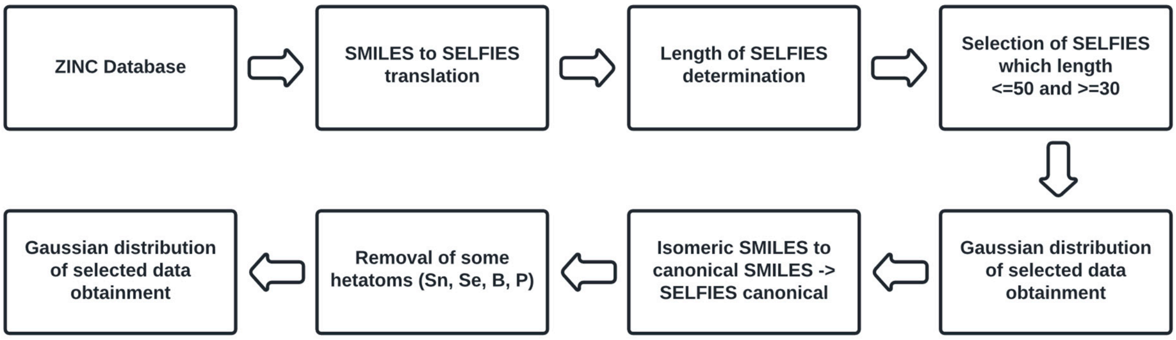

Figure S1. Data for model training preparation.

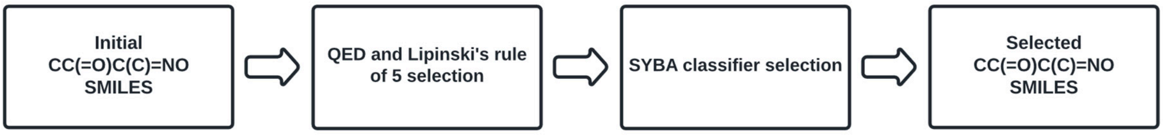

Figure S2. Initializer selection workflow.

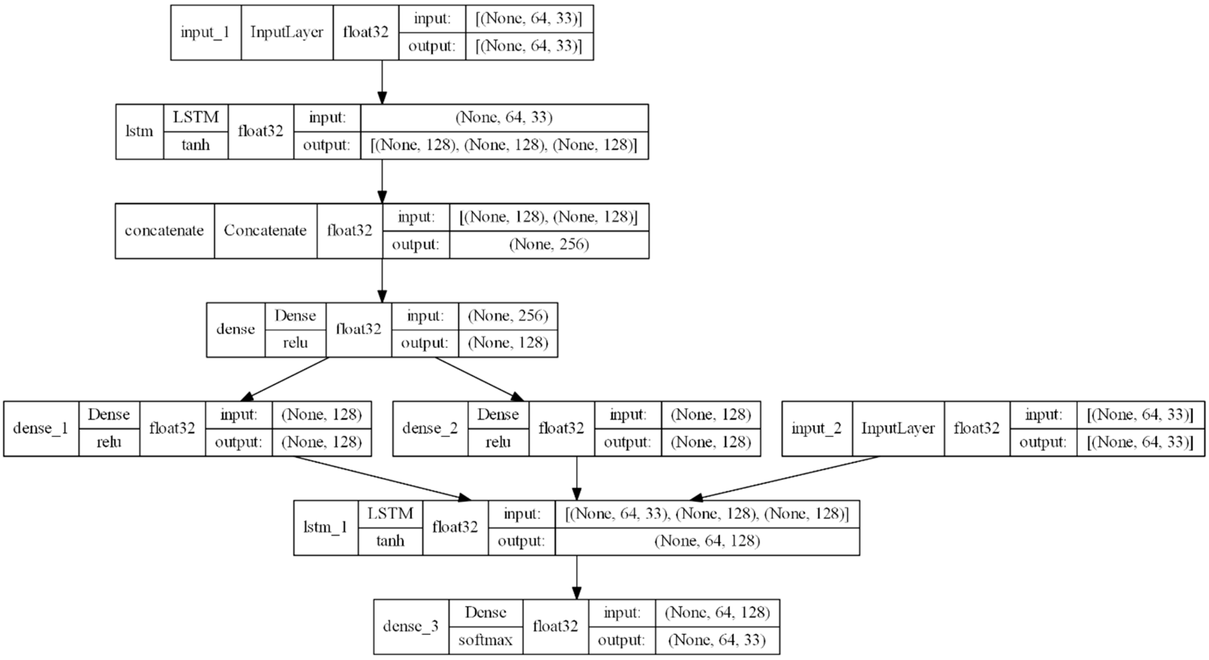

Figure S3. The seq_to_seq model architecture.

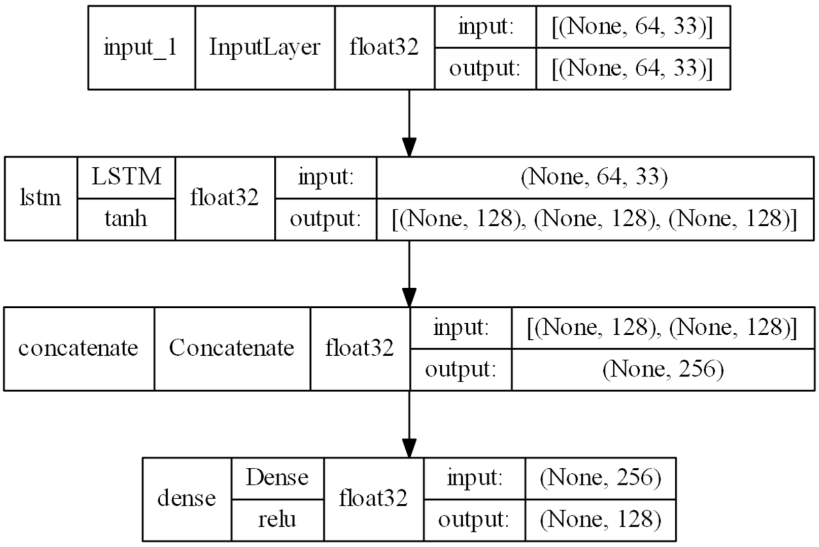

Figure S4. Part of the model that encodes molecular sequences into latent space (see **File S16**).

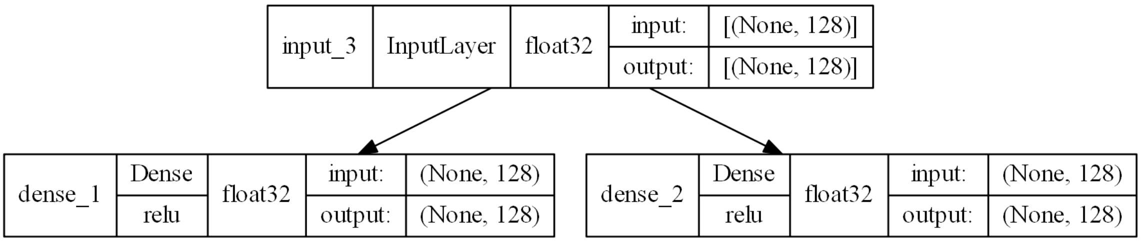

Figure S5. The latent to states model takes the tensor of a given dimension and as a result, states are decoded (see **File S17**).

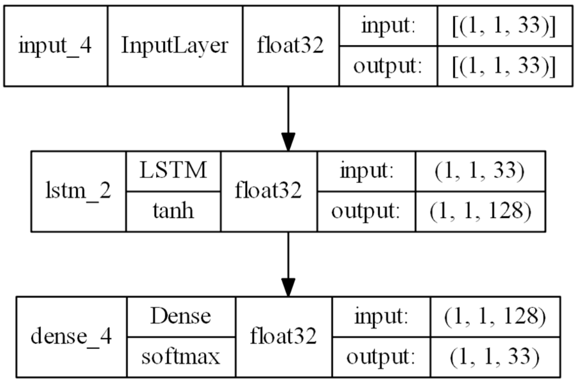

Figure S6. The sample model is used to make predictions, character by character (see **File S18**).

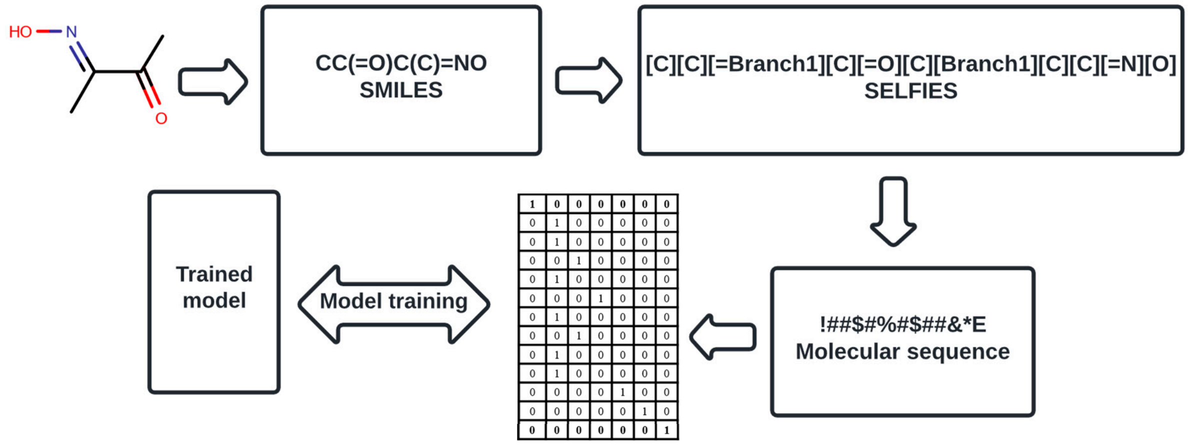

Figure S7. The overall training workflow.

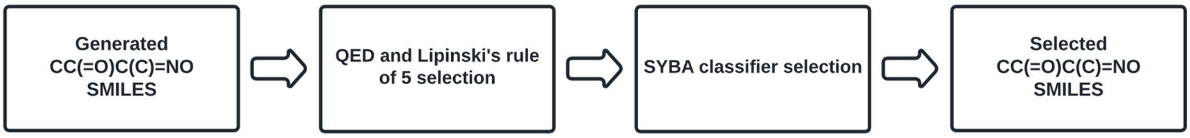

Figure S8. Selection of generated structures workflow.

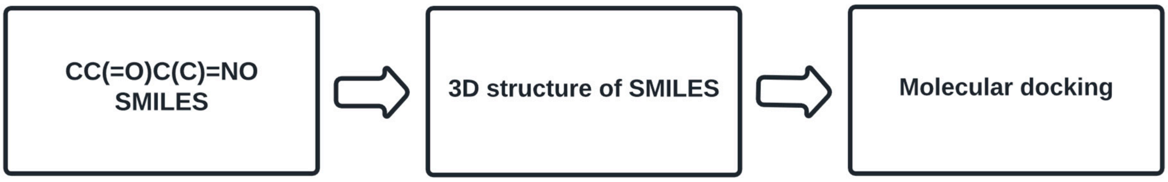

Figure S9. Workflow for molecular docking.

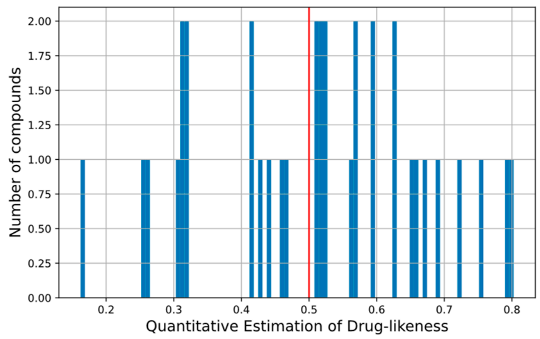

Figure S10. The QED distribution histogram for ROR-γ active compounds with a marked threshold (red vertical line).

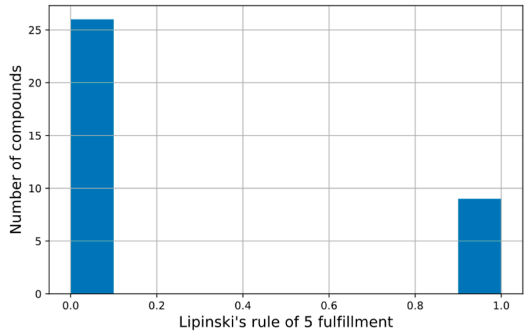

Figure S11. Histogram of Lipinski's rule of 5 fulfillment distribution for ROR-γ active compounds.

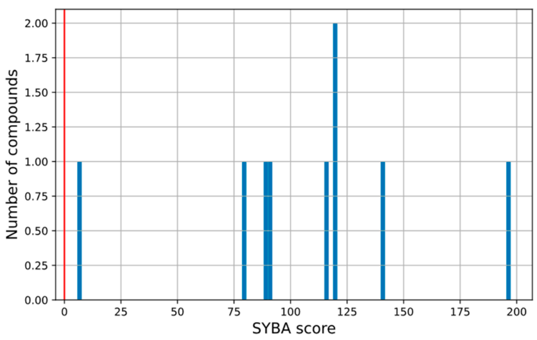

Figure S12. The SYBA score distribution histogram for ROR-γ active with a marked threshold (red vertical line).

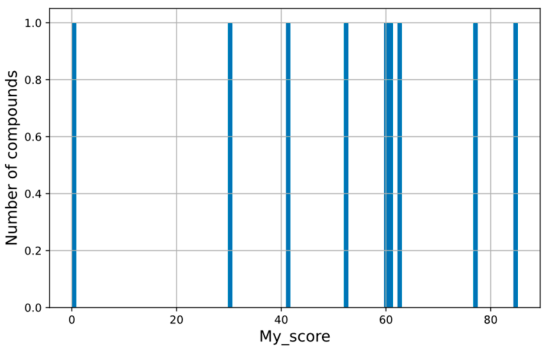

Figure S13. My score distribution to 9 structures that passed through three filters.

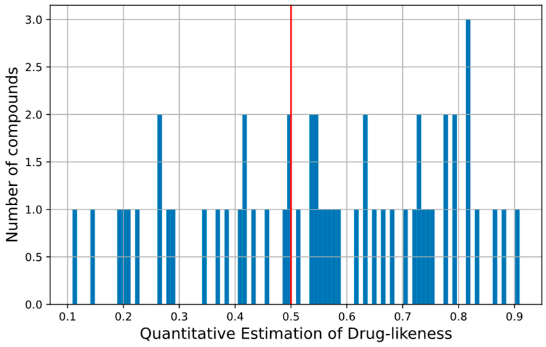

Figure S14. The QED distribution histogram for the first prediction with a marked threshold (red line).

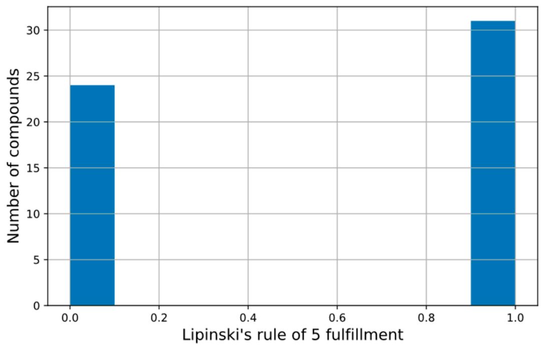

Figure S15. Histogram of Lipinski's rule of 5 fulfillment distribution for the first prediction.

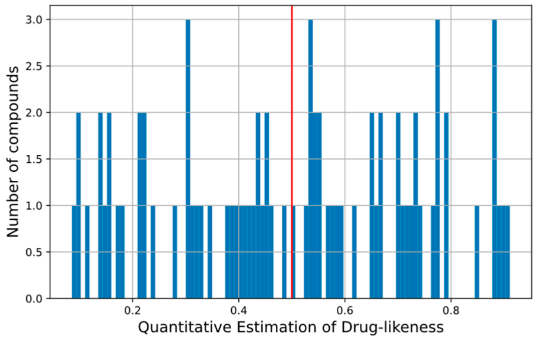

Figure S16. The QED distribution histogram for the second prediction with a marked threshold (red vertical line).

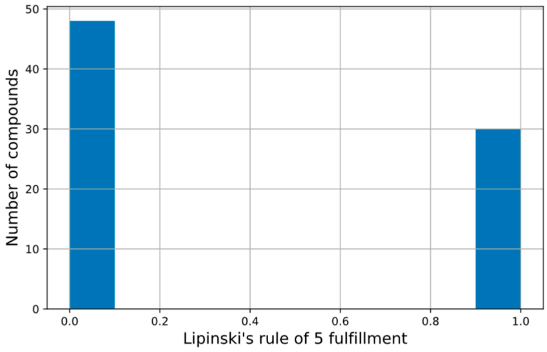

Figure S17. Histogram of Lipinski's rule of 5 fulfillment distribution for the second prediction.

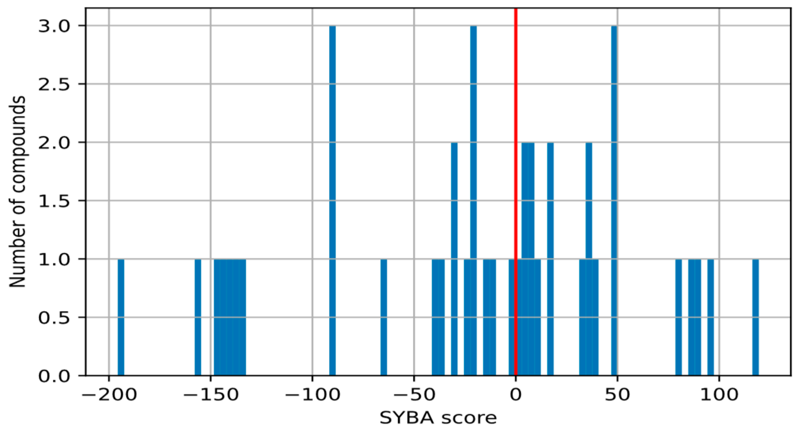

Figure S18. The SYBA score distribution over forty-two selected structures with a marked threshold (red vertical line).

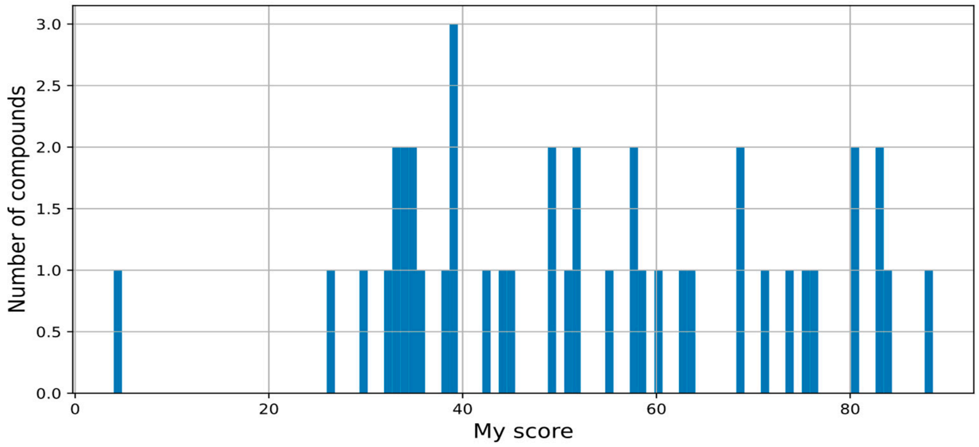

Figure S19. My score distribution for **File S29**. – all generated SMILES (forty-two structures).

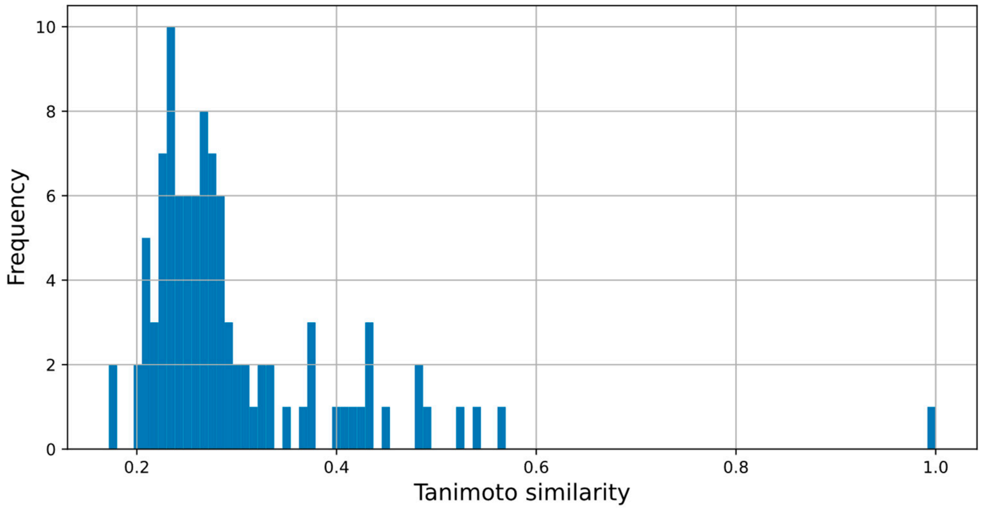

Figure S20. The Tanimoto similarity distribution along with twenty generated structures after selection (see **Table 1.**) and five initials.

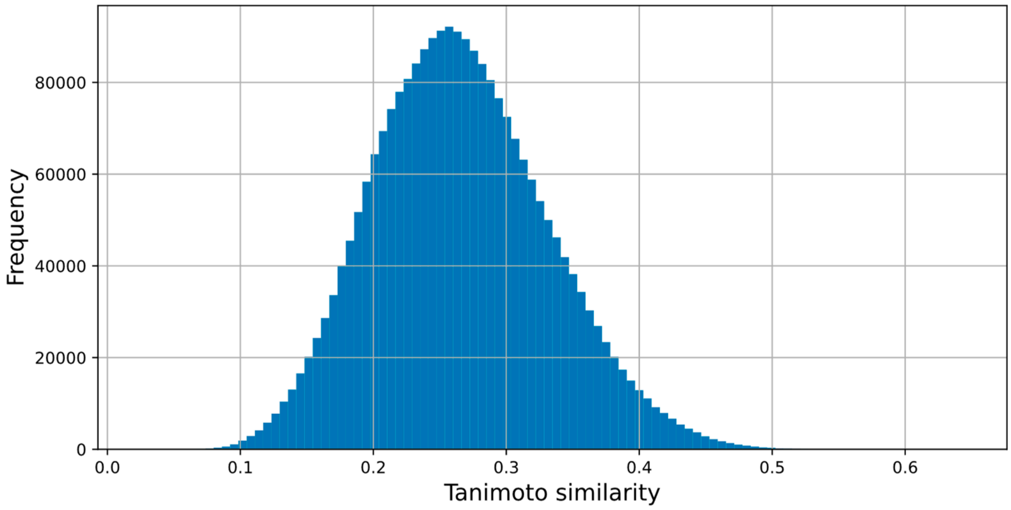

Figure S21. The Tanimoto similarity distribution histogram between “to be docked” molecules and the training data.

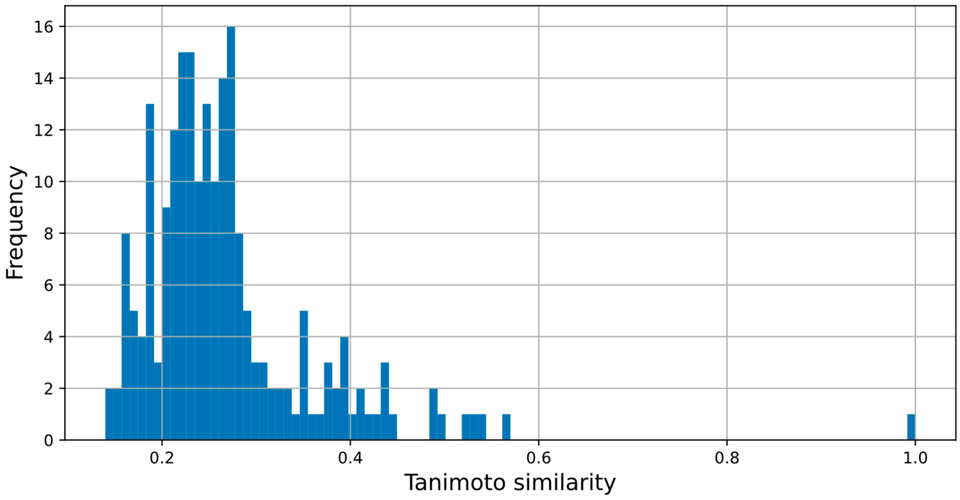

Figure S22. The Tanimoto similarity distribution between data after QED, Lipinski’s rule of five selections, and initials.

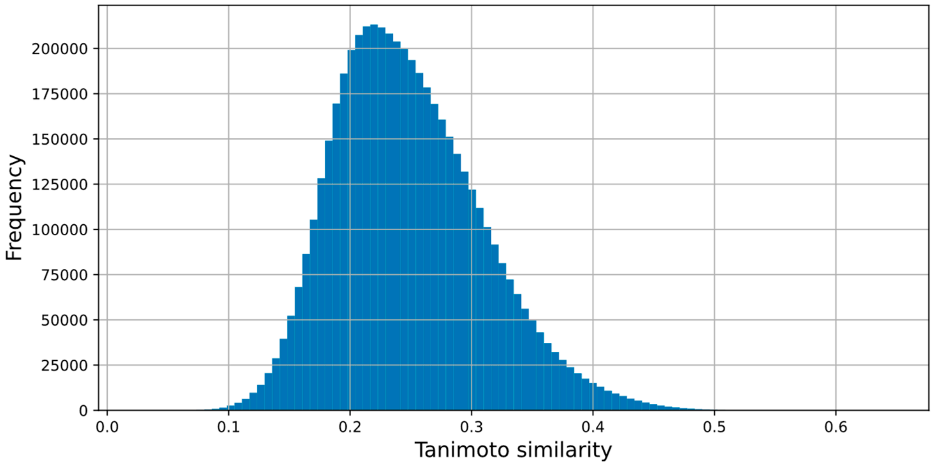

Figure S23. The Tanimoto similarity distribution histogram between forty-two structures and training data.
