# Supplementary material for: Neural Networks in the Design of Molecules with Affinity to Selected Protein Domains
Source: Int J Mol Sci. 2023 Jan 16;24(2):1762. doi: 10.3390/ijms24021762 (PMC9865393; doi:10.3390/ijms24021762)
Supplement: Supplementary file 1 [file ijms-24-01762-s001.zip › Files.pdf]

Files

File S1.	ZINC-downloader-2D-smi.wget
File S2.	Data_preparation.ipynb
File S3.	zinc20_selected_to_create_model_processed.parquet
File S4.	RORgamma_active_compounds.xlsx
File S5.	ROR_gamma_active_QED_Lipinski.ipynb
File S6.	ROR_gamma_active_QED_Lipinski.xlsx
File S7.	SYBA_class_ROR_gamma_activ_AFTER_QED_LIPINSKI.ipynb
File S8.	Prediction_initializers_ROR_activ.xlsx
File S9.	SELFIES_coder.py
File S10.	UniCode_char.csv
File S11.	Molecule_generator-Generative_neural_network.py
File S12.	SELFIES_to_mol_seq.json
File S13.	mol_seq_to_SELFIES.json
File S14.	mol_seq_to_int.json
File S15.	int_to_mol_seq.json
File S16.	mol_seq2lat_ZINC.h5
File S17.	lat2state_ZINC.h5
File S18.	samplemodel_ZINC.h5
File S19.	Prediction_1_0.1_tensor_scaling.ipynb
File S20.	Prediction_2_0.2_tensor_scaling.ipynb
File S21.	Molecules_generated_tensor_scaling_0_1.xlsx
File S22.	Molecules_generated_tensor_scaling_0_2.xlsx
File S23.	Selection_from_0_1_tensor_scaling.ipynb
File S24.	Selection_from_0_2_tensor_scaling.ipynb
File S25.	Selected_molecules_from_0_1_tensor_scaling.xlsx
File S26.	Selected_molecules_from_0_2_tensor_scaling.xlsx
File S27.	Combine_Generated_and_Selected_structures.ipynb
File S28.	Assigning_prediction_mode_to_selected_SMILES.ipynb
File S29.	All_generated_SMILES_QED_Lipinski.xlsx

File S30.	SYBA_classifier_additional_filter.ipynb
File S31.	All_generated_SMILES_SYBA_filtration.xlsx
File S32.	All_generated_SMILES_visualization.ipynb
File S33.	Python_molecular_docking.py
File S34.	Tanimoto_similarity-SYBA_selection.ipynb
File S35.	Tanimoto_similarity_All_generated_and_selected.ipynb
File S36.	Checking_if_ROR_gamma_activ_are_in_ZINC_db.ipynb
File S37.	dockingResults_ROR_gamma_SYBA_selected.xlsx
File S38.	Clean_results.ipynb
File S39.	Selection_of_most_prominent_structures.ipynb
File S40.	SMILES_to_3D_PDB.ipynb
File S41.	My_score_to_final_structures.ipynb
File S42.	dockingResults_ROR_gamma_SYBA_CLEAN.xlsx
File S43.	Reference_dockings folder
File S44.	7npc_structure_of_native_ligand.dlg
File S45.	7NP5_structure_of_native_ligand.dlg
File S46.	7KXD_structure_of_native_ligand.dlg
File S47.	master_thesis_calculations.xlsx
